# Production of l(+)-lactic acid from acid pretreated sugarcane bagasse using *Bacillus coagulans* DSM2314 in a simultaneous saccharification and fermentation strategy

**DOI:** 10.1186/s13068-016-0646-3

**Published:** 2016-11-15

**Authors:** Edwin C. van der Pol, Gerrit Eggink, Ruud A. Weusthuis

**Affiliations:** 1 Bioprocess Engineering, Wageningen University and Research, PO Box 16, 6700 AA Wageningen, The Netherlands; 2 Food and Biobased Research, Wageningen University and Research, PO Box 17, 6700 AA Wageningen, The Netherlands

**Keywords:** Fermentation, Lactic acid, Simultaneous saccharification and fermentation (SSF), Enzymatic hydrolysis, Bagasse, Lignocellulose

## Abstract

**Background:**

Sugars derived from lignocellulose-rich sugarcane bagasse can be used as feedstock for production of l(+)-lactic acid, a precursor for renewable bioplastics. In our research, acid-pretreated bagasse was hydrolysed with the enzyme cocktail GC220 and fermented by the moderate thermophilic bacterium *Bacillus coagulans* DSM2314. Saccharification and fermentation were performed simultaneously (SSF), adding acid-pretreated bagasse either in one batch or in two stages. SSF was performed at low enzyme dosages of 10.5–15.8 FPU/g DW bagasse.

**Results:**

The first batch SSF resulted in an average productivity of 0.78 g/l/h, which is not sufficient to compete with lactic acid production processes using high-grade sugars. Addition of 1 g/l furfural to precultures can increase *B. coagulans* resistance towards by-products present in pretreated lignocellulose. Using furfural-containing precultures, productivity increased to 0.92 g/l/h, with a total lactic acid production of 91.7 g in a 1-l reactor containing 20% W/W DW bagasse. To increase sugar concentrations, bagasse was solubilized with a liquid fraction, obtained directly after acid pretreatment. Solubilizing the bagasse fibres with water increased the average productivity to 1.14 g/l/h, with a total lactic acid production of 84.2 g in a 1-l reactor. Addition of bagasse in two stages reduced viscosity during SSF, resulting in an average productivity in the first 23 h of 2.54 g/l/h, similar to productivities obtained in fermentations using high-grade sugars. Due to fast accumulation of lactic acid, enzyme activity was repressed during two-stage SSF, resulting in a decrease in productivity and a slightly lower total lactic acid production of 75.6 g.

**Conclusions:**

In this study, it is shown that an adequate production of lactic acid from lignocellulose was successfully accomplished by a two-stage SSF process, which combines acid-pretreated bagasse, *B. coagulans* precultivated in the presence of furfural as microorganism, and GC220 as enzyme cocktail. The process may be further improved by enhancing enzyme hydrolysis activities at high lactic acid concentrations.

**Electronic supplementary material:**

The online version of this article (doi:10.1186/s13068-016-0646-3) contains supplementary material, which is available to authorized users.

## Background

Lactic acid is conventionally used as natural preservative in food and cosmetics. A relatively new application is its utilization as intermediate for the production of chemicals and polymers [1]. Polymerized lactic acid (PLA) can be used as bioplastic, serving as a biobased alternative to oil-derived plastics such as polyethylene and polystyrene [1]. Lactic acid can also be further converted into acrylic acid, an intermediate used in the plastic and textile industry [2].

Lactic acid is commonly produced in bacterial fermentation processes, using high-grade sugar or sugar-rich resources such as molasses or starch as feedstock [3]. However, due to the limited availability of sugar-rich crops, exploration of alternative feedstocks is a main target of current research [4]. Lignocellulose, consisting of 60–75% polymerized sugar on weight basis, is an interesting alternative feedstock [5]. In this study, sugarcane bagasse is used as source of lignocellulose.

A combination of thermo-chemical pretreatment and enzymatic hydrolysis can be used to obtain fermentable monomeric sugars from sugarcane bagasse lignocellulose [6, 7]. However, thermo-chemical pretreatment also results in the formation of by-products such as organic acids, phenolics and furans. These compounds can negatively influence microbial growth and product formation during fermentation [6, 8].

**Fig. 1:**
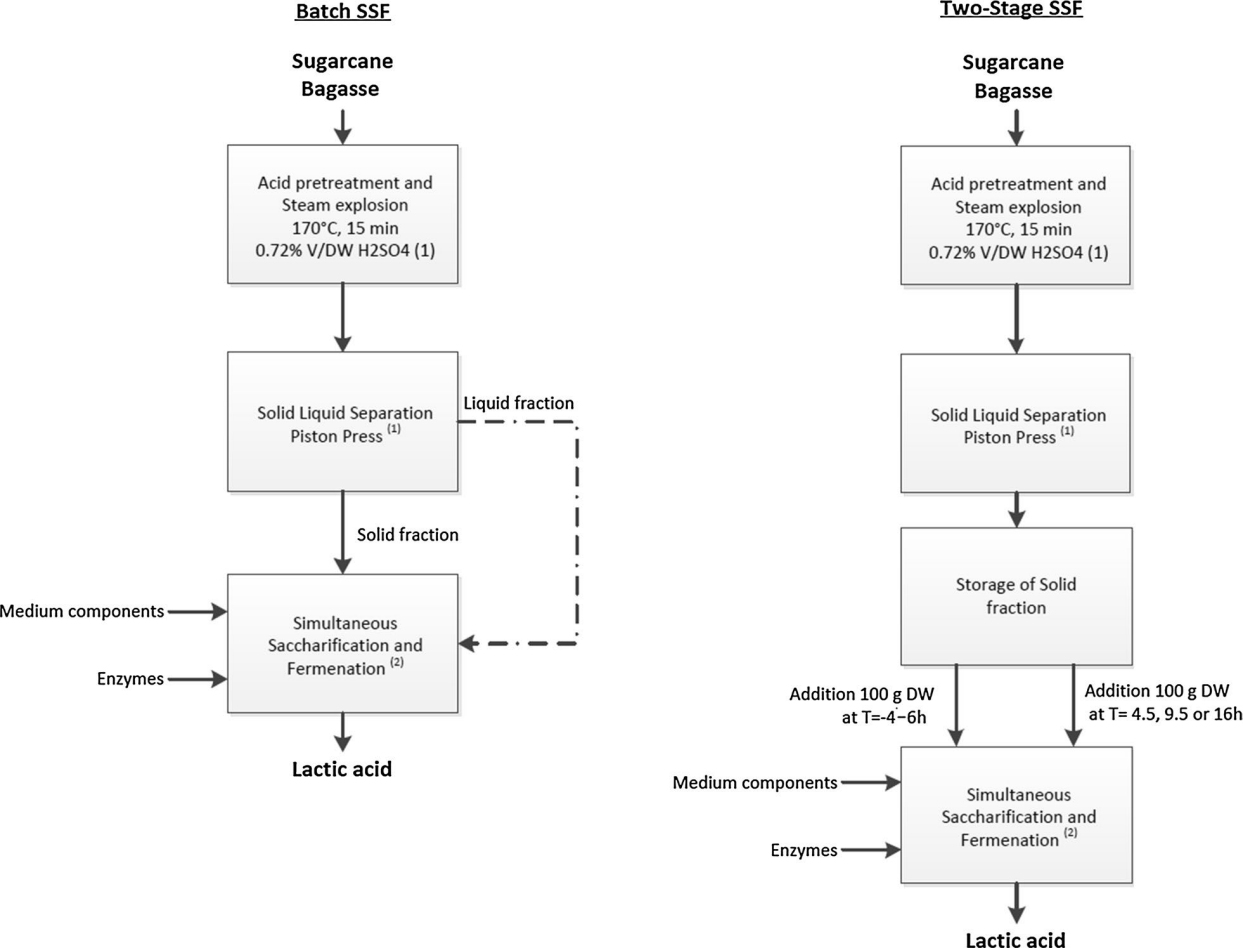
Process overview of the production of lactic acid from sugarcane bagasse, either using batch SSF or two-stage SSF. *1*: acid pretreatment on pilot plant scale as shown in described research [7]. *2*: Simultaneous saccharification and fermentation (SSF) of the solid fraction obtained after solid–liquid separation (this work). The liquid fraction can either be used in the SSF, or can be used in a separate fermentation

After thermo-chemical pretreatment, a solid–liquid separation is performed (Fig. 1). The solid fraction contains bagasse fibres which consist mainly of cellulose, while part of the hemicellulose and lignin may also be present in this fraction [5, 7]. The liquid fraction contains some hemicellulose-derived oligomeric and monomeric sugars, while also a part of the lignin can be present in this liquid fraction.

Pretreated bagasse fibres have a high water-holding capacity, resulting in a viscous broth at high dry weight (DW) concentrations [7]. Therefore, a maximum of 20% can be added to the fermentation before the broth becomes too viscous. Since bagasse has a DW of 31% W/W after thermo-chemical pretreatment, it has to be diluted before it can be hydrolysed and fermented. Sugar concentrations can be increased by solubilizing the bagasse fibres with the liquid fraction, resulting from solid–liquid separation of pretreated biomass (Fig. 1).

Separate hydrolysis and fermentation (SHF) is an often described process to monomerize (hemi)cellulosic sugars, and to ferment those sugars to lactic acid in two separate steps [9]. During enzymatic hydrolysis, increasing concentrations of sugars inhibit enzymatic activity, making it difficult to efficiently obtain high sugar concentrations [10]. In simultaneous saccharification and fermentation processes (SSF), enzymes and microorganisms are simultaneously active in the same reactor. This reduces product inhibition on enzymatic activity, since released monomeric sugars are directly consumed by the microorganism [11]. Conditions applied in SSF processes such as pH and temperature should be suitable for both the enzyme cocktail and microorganism.

*Bacillus coagulans* is an interesting strain for the production of lactic acid from lignocellulose in an SSF process [11]. It can ferment both glucose and xylose homo-fermentatively with conversion efficiencies of over 90% W/W. Furthermore, *B. coagulans* produces lactic acid with a high productivity of 2.5–3 g/l/h [12–14]. It is able to grow in slightly acidic environments while it is also a moderate thermophile with an optimum growth temperature of approximately 50 °C, similar to optimal conditions for commercial enzyme cocktails such as GC220 (Genencor, Denmark) and CTeC2 (Novozymes, Denmark). However, growth of *B. coagulans* can be inhibited by by-products present in pretreated lignocellulose [8]. A previous study has shown that the inhibitory effects of by-products can be reduced by adding low concentrations of furfural, one of the inhibitory by-products found in acid-pretreated bagasse, to the preculture of *B. coagulans* [15]. Addition of 1 g/l furfural to precultures of *B. coagulans* increased lactic acid productivity, yield and titre in fermentation processes [15]. Although these findings can be of interest for fermentation of pretreated lignocellulosic material, they have only been observed in a theoretical approach, where an artificial substrate resembling the composition of acid-pretreated bagasse was used. Therefore, in this paper, it is investigated whether furfural addition to precultures of *B. coagulans* also has a beneficial effect on fermentation processes using actual acid-pretreated bagasse as substrate. Furthermore, other challenges of the SSF process such as viscosity are tackled in this study. To obtain an SSF process which is economically attractive, and which can compete with lactic acid production on high-grade sugars, lactic acid productivities and yields similar to fermentations on high-grade sugars should be reached, using minimal amounts of enzyme.

## Methods

### Chemicals, enzymes and biomass

Most chemicals used were ordered at Sigma-Aldrich (St. Louis, USA), and had a purity of at least 98%. Exceptions were yeast extract, peptone, glucose and bis–tris, which were ordered at Duchefa (The Netherlands).

The enzyme cocktail used in most processes was Genencor GC220, which had an activity of 105 FPU/ml (Genencor, Denmark) [16]. In one experiment, Novozymes CTeC 2 was used, which had an activity of 168 FPU/ml (Novozymes, Denmark).

Sugarcane was harvested in Queensland, Australia. The bagasse residue after sugar extraction was pretreated for 15 min at 170 °C using 0.72% sulphuric acid, followed by steam explosion, as described previously [7]. The final solid material contained 47% glucan and 3% xylan.

### Microorganism

*Bacillus coagulans* DSM 2314 was acquired as freeze-dried stock at the German collection of microorganisms and cell cultures (DSMZ, Germany). Cells were suspended for 30 min in 5 ml PYPD medium, consisting of 5 g/l yeast extract, 10 g/l peptone, 20 g/l glucose and 10 g/l bis–tris, pre-sterilized for 20 min at 121 °C. Cells were transferred to 60-ml anaerobic flasks containing 50 ml PYPD medium, and grown for 16 h to reach an optical density measured at 660 nm (OD 660) of approximately 2. After addition of glycerol to reach a concentration of 15% v/v in the sample, cells were stored in 1.5-ml aliquots in cryovials at −80 °C until used.

### Preculture preparation

Sixty millilitre sterile anaerobic flasks were filled with either 50 ml PYPD medium for reference precultures, or 25 ml 2× concentrated PYPD medium sterilized at 121 °C for 20 min, and 25 ml of 2 g/l furfural dissolved in milliQ water, which was pasteurized for 1 h at 85 °C, for furfural-containing precultures. The anaerobic flasks were inoculated with 250 µl *B. coagulans* freezer stock to obtain a starting OD660 of 0.01, and cultivated in an incubator at 50 °C without shaking. When an OD_660_ of 1 was reached, the preculture was added as inoculum to the SSF process.

### Batch simultaneous saccharification and fermentation (SSF)

Fermentation experiments were performed in Multifors reactors (Infors, Switzerland), which were pre-sterilized empty for 20 min at 121 °C. The SSF consisted of two phases, a pre-hydrolysis phase of 4–6 h, and a fermentation phase. During the pre-hydrolysis phase, 645 g of pretreated solid bagasse (31% W/W dry weight), corresponding to 200 g of dry weight, was added to the fermenter, together with 245 ml of sterile water or simulated liquid fraction, of which the composition was based on previous findings [7] (Table 1). Also, 25% of the total amount of enzymes was added. The pH was set to 5.0 and controlled by addition of 4 N Ca(OH)_2_ (14.8% W/V). The temperature of the fermenter was set at 50 °C, the stirrer speed was initially started at 40 RPM, and increased to 100 RPM during the 1st h when the viscosity of the biomass was sufficiently decreased, and no active aeration was applied. After the pre-hydrolysis phase, 100 ml of a 100 g/l of yeast extract solution was added to the biomass, and the fermenter was inoculated with 50 ml of preculture (5% V/V).

**Table 1:** Composition of the liquid fraction acquired after solid–liquid separation of acid-pretreated sugarcane bagasse, as determined in a previous study [7]

By-product	Presence (g)
Xylose	3.98
Glucose	1.15
Acetic acid	0.521
Furfural	0.272
Glycolic acid	0.165
Coumaric acid	0.064
Formic acid	0.052
Vanillin	0.016

The presence of different compounds is shown in gram per 245 ml of liquid fraction

*Bacillus coagulans* has an optimal growth at a temperature of 54 °C and a pH of 6.5, but can grow at a broad temperature range between 30 and 58 °C, and pH range of 4.8–7.5 [11, 15]. Hydrolysis of pretreated bagasse was achieved by the enzyme cocktail Genencor GC220, which has a temperature optimum of 50 °C and a pH optimum at 5.0. At pH 5.8 the conversion rate of glucan to glucose by GC220 is at 85% of the conversion rate at pH 5. As conditions for the SSF processes, 50 °C and pH 5.8 were chosen.

During fermentation, the stirrer speed was set at 100 RPM, the temperature was controlled at 50 °C and the pH was controlled at 5.8 by the addition of 4 N Ca(OH)_2_ (14.8% W/V). The total amount of GC220 enzyme cocktail added during SSF was 10% V/DW (corresponding to 10.5 FPU per g DW material), 12.5% v/dw (corresponding to 13.1 FPU per g DW material) or 15% V/DW (corresponding to 15.8 FPU per g DW material). At regular intervals during all SSF experiments, samples of 10 ml were taken and stored at −20 °C until analysis was performed.

### Two-stage simultaneous saccharification and fermentation (SSF)

The pre-hydrolysis phase of the two-stage SSF experiment was started with the addition of 322.5 g of pretreated bagasse, equivalent to 100 g dry weight, 245 ml of 20.4 g/l (NH_4_)_3_PO_4_ in sterile water, and 25% of the total enzymes used. The pH was initially set to pH 5 by addition of 4 N H_2_SO_4_ or 4 N Ca(OH)_2_, and was controlled afterwards by addition of 4 N Ca(OH)_2_. Temperature was kept at 50 °C, and stirring was kept at 100 RPM. The inoculation procedure after pre-hydrolysis was similar to the batch SSF. At 4.5 h or 9.5 h, 322.5 g of additional pretreated bagasse was added gradually over the time span of 1.5 h, while the stirring speed was increased to 150 RPM. In total, 15% V/DW enzymes was added, corresponding to 15.8 FPU/g DW pretreated bagasse.

### Analysis of lactic acid and monomeric sugars

Analysis of lactic acid and sugars was performed using a Waters e2695 HPLC system (Milford, USA) equipped with a Waters Refractive index RI2414 and a Waters 2489 UV/Vis spectrophotometer measuring at 210 nm. The column used was a Shodex RS pak KC-811 ion exchange column (length 300 mm—I.D. 8 mm), controlled at 65 °C. As eluent, 3 mM H_2_SO_4_ in milliQ water was used. The flow used was 1 ml/min.

Samples taken during SSF were thawed and vortexed. 1.5 ml of sample was transferred to a 2 ml Eppendorf tube and placed in a heating block for 15 min at 70 °C, ensuring all calcium lactate was dissolved. Samples were centrifuged for 4 min at 13,200 RPM. 250 µl of supernatant was mixed with 250 µl of water and 500 µl of 1 M H_2_SO_4_/1 mM phthalic acid (which was used as internal standard) in milliQ water. Samples were heated again to 70 °C for 15 min, cooled down for 2 h, filtered using 0.2 µm Spartan filters to ensure all calcium was precipitated and removed, and supernatants were measured using HPLC.

### Glucan, xylan and uronic acid analysis

The neutral sugar content and composition was determined in duplicate according to Englyst and Cummings [17]. After pre-hydrolysis with 72% (w/w) H_2_SO_4_ for 1 h at 30 °C, the samples were hydrolysed with 1 M H_2_SO_4_ at 100 °C for 3 h. The monosaccharides were derivatized to their alditol acetates and analysed by gas chromatography (Focus-GC, Thermo Scientific, Waltham, MA, USA). Inositol was used as internal standard.

The uronic acid content was determined in duplicate according to the automated colorimetric *m*-hydroxydiphenyl assay [18], using an auto-analyser (Skalar Analytical B.V., Breda, The Netherlands). Galacturonic acid was used for calibration.

### Effect of lactic acid concentration on enzymatic hydrolysis

150-ml shake flasks were filled with 6.5 g of acid-pretreated bagasse, corresponding to 2 g of DW. To these shake flasks, 15 ml 0.1 M citric acid and 23 ml 0.2 M Na_2_HPO4.12H_2_O was added to obtain a buffer at pH 5.8. To these flasks, 0, 1.25, 2.5 and 5 ml of (d/l)-lactic acid solution (80% in H_2_O, density 1.2 g/ml) was added. MilliQ water was used to set the final working volume at 50 ml. The pH was reset to 5.8 using KOH pellets when required. The shake flasks were incubated for 15 min at 50 °C and 100 RPM. After addition of 100 µl of the enzyme cocktail GC220, the flasks ware incubated at 50 °C and 200 RPM. After 1 and 2 h, samples were taken, immediately heated to 98 °C for 10 min to inactivate the enzymes, cooled on ice, filtered using 0.2 µm Spartan filters, and stored at 4 °C. Glucose concentrations were measured using the previously described HPLC method.

### Calculations determining lactic acid production, yields and sugar monomerization

Due to a large increase in volume during the SSF, values were recalculated to gram per batch instead of gram per litre to determine yields and total lactic acid produced. Volume of the reactor at time point t (*V*_R*,t*_) was calculated based on the amount of base added in percentage of the total amount of base added at time point t (*B*_t_), volumes of the reactor at *T* = 0 in litre (*V*_*F,0*_), determined volume of the reactor at the end of the SSF in litre (*V*_F,end_), and the total volume of sample taken at time point t in litre (*V*_S_). The amount of lactic acid in gram at time point t (*A*_LA*,t*_) was calculated based on the concentration of lactic acid in gram per litre determined via HPLC at time point t (*C*_LA*,t*_), the volume of the reactor at time point t(*V*_R,t_), and a correction factor for the amount of lactic acid taken out by sampling at sample n (CF_LA*,n*_).

$$A_{{{\text{LA}},t}} = C_{{{\text{LA}},t}} * V_{{{\text{R}},t}} + {\text{CF}}_{{{\text{LA}},n}}$$ with $$V_{{{\text{R}},t}} = V_{{{\text{F}},0}} + \frac{{B_{t} }}{{B_{\text{end}} }} * \left( {V_{{{\text{F}},\,{\text{end}}}} - V_{{{\text{F}},0}} } \right) - V_{\text{S}}$$ and $${\text{CF}}_{{{\text{LA}},n}} = \sum\nolimits_{i = 1}^{n - 1} {C_{{{\text{LA}},i}} *\,\,V_{{{\text{S}},i}} }$$

The solid fraction contained 47% glucans and 3% xylans when added to the reactor. Due to the monomerization reaction being a hydrolysis reaction, the molecular weight increases from 162 g/mol of subunit glucan to 180 g/mol glucose, and from 132 g/mol of subunit xylan to 150 g/mol xylose, thus the maximum amount of monomeric sugars which can be formed from 200 g of dry weight solid material is 110.2 g of sugars.

During earlier experiments [7], and by residual glucan/xylan analysis at the end of the SSF (Table 4), it was observed that from 200 g of pretreated solid biomass, around 92 g of sugars were monomerizable by the enzyme cocktail and were thus available for fermentation.

Lactic acid production can be estimated based on the amount of Ca(OH)_2_ added to the reactor. Lactic acid concentration at a certain time (*A*_LA*,t*_) was calculated by taken the amount of base added at a certain timepoint (*B*_*t*_) divided by the total amount of base added (*B*_*t = end*_), and multiplied by the final lactic acid titre (*A*_LA,t*=end*_) measured in triplicate via HPLC. The overall calculation is:

$$A_{{{\text{LA}},t}} = \frac{{B_{t} }}{{B_{{t = {\text{end}}}} }} * A_{{{\text{LA}},t = {\text{end}}}}$$

No difference in lactic acid production larger than 2 g was observed between the calculations based on the Ca(OH)_2_ addition, and HPLC analysis of samples taken at different time points during the SSF.

## Results and discussion

### Batch SSF, solubilizing bagasse fibres with liquid fraction

Since lactic acid is a bulk chemical with low added value, the process requires an efficient conversion of lignocellulosic material into lactic acid with high productivities, yields and titres [19]. The dry matter content of bagasse fibres after pretreatment and solid–liquid separation was 31% W/W. A bagasse fibre concentration over 20% (W/W, dry weight basis) results in too high viscosities in the fermenter. Therefore, the fibre fraction was diluted by adding 245 ml of liquid fraction, acquired during solid–liquid separation performed directly after thermo-chemical pretreatment.

In this batch SSF process, a total lactic acid production of 77.6 g was obtained in a fermenter with 1 l starting volume, with a final lactic acid concentration of 64.1 g/l. The average lactic acid productivity was 0.78 g/l/h, which is low compared to the 2.5–3 g/l/h observed for lactic acid fermentation processes using high-grade sugars [12–14] (Table 3). The conversion yield on total lignocellulosic sugars to lactic acid was 74% W/W, whereas the conversion yield on monomerizable sugars was 80% W/W. Since both the average productivity and yield are not sufficient to compete with processes using pure sugars, the SSF process should be optimized to increase lactic acid productivity on the lignocellulosic material.

### Batch SSF inoculated with *B. coagulans* preculture containing furfural

In earlier research, it was found that the addition of furfural to precultures of *B. coagulans* improved growth and lactic acid production on substrates rich in lignocellulosic by-products [15]. However, this research was performed using a model substrate. Although the composition of this substrate resembles acid-pretreated bagasse with regard to presence of by-products, other process parameter such as increased viscosity during SSF, and the presence of compounds like lignin are not taken into account. The effect of furfural addition to precultures was therefore also investigated in an SSF set-up using bagasse fibres solubilized with liquid fraction.

**Fig. 2:**
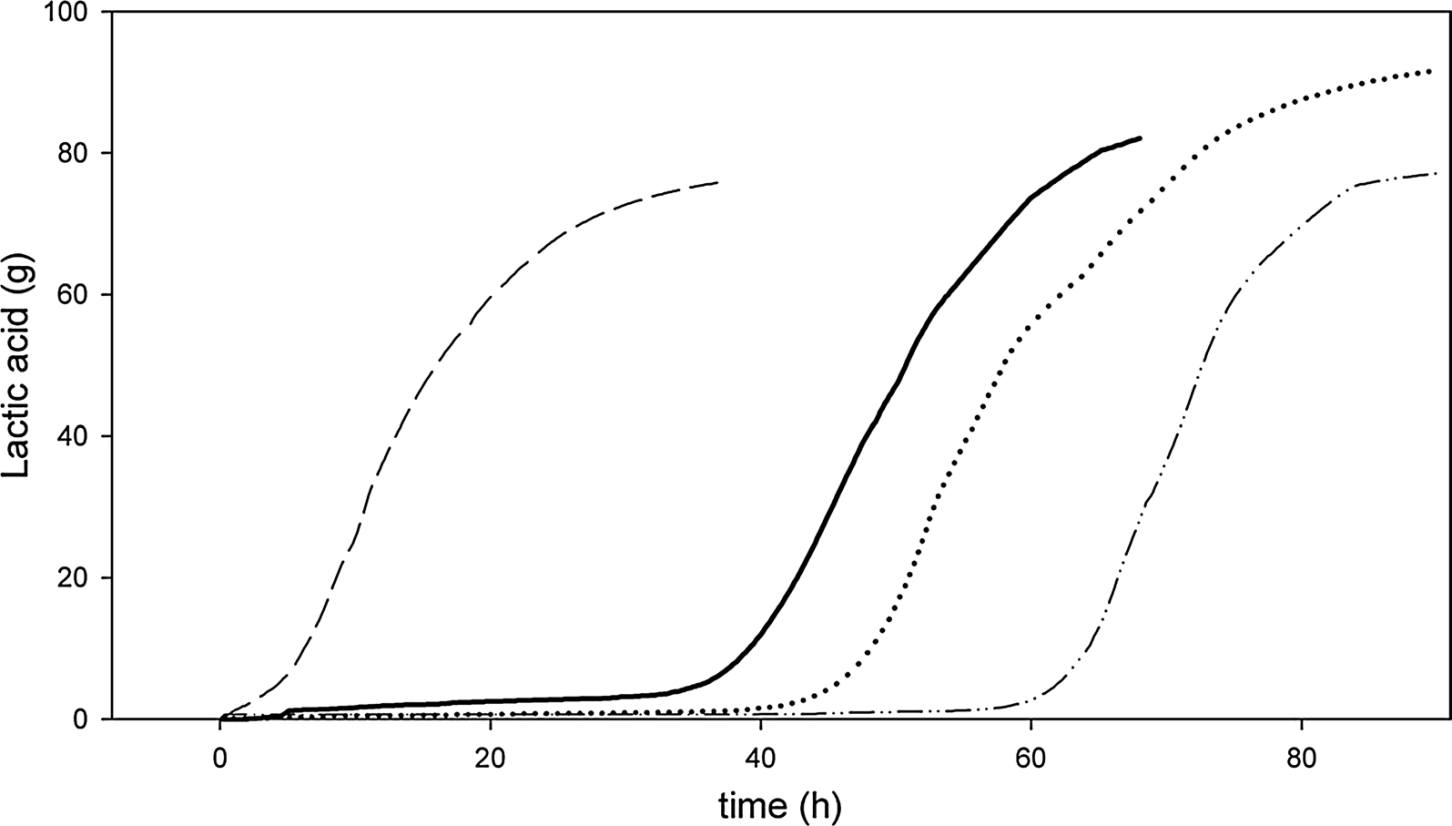
Simultaneous saccharification and fermentation (SSF) experiments performed using 200 g/l acid-pretreated bagasse DW and 10.5–15.8 FPU/g DW bagasse GC220 enzyme cocktail in pH controlled fermentations with a start volume of 1 l. As inoculum, either furfural-containing or reference preculture was used. The bagasse was either added in one batch or in two stages. Lactic acid production followed by calcium hydroxide addition, verified with HPLC measurements. The following experiments were performed: batch SSF solubilized with liquid fraction, inoculated with reference preculture (*dashed line* with *dots*), batch SSF solubilized with liquid fraction, inoculated with furfural preculture (*dotted line*), batch SSF solubilized with water, inoculated with furfural preculture (15% V/DW enzymes) (*straight line*), two-stage SSF solubilized with water, inoculated with furfural preculture (*dashed line*)

Addition of 1 g/l furfural to the preculture, used as inoculum for the batch SSF experiment, reduced the initial lag phase by 20 h (Table 2; Fig. 2). Besides the reduction in lag phase, the yield of lactic acid on total lignocellulosic sugars was 87% W/W when a furfural preculture was used, which is significantly higher than the yield of 74% W/W observed when a reference preculture was used. Furfural addition increased total lactic acid production produced in the fermenter with 1 l starting volume by 18% to 91.7 g, while average lactic acid productivity increased with 0.14–0.92 g/l/h. The maximum lactic acid productivity was 4.2 g/l/h.

**Table 2:** SSF experiments performed with 20% DW pretreated bagasse using the enzyme cocktail GC220 at different enzyme dosages to monomerize sugars, and *B. coagulans* DSM2314 for fermentation to lactic acid

SSF	Biomass liquefied with	Enzyme added %V/DW	Preculture	*C*_La_ (g/l)	*A*_La_ (g)	Q_v,av_ (g/l/h)	*Q*_*v*,max_ (g/l/h)	*Y*_s/La_ (%)	*Y*_Lc/La_ %	Time (h)^a^
Batch	Liquid fraction	12.5	Reference	64.1	77.6	0.78	4.2	80	74	90
Batch	Liquid fraction	12.5	Furfural	74.6	91.7	0.92	4.2	94	87	90
Batch	Water	7.5–>10	Furfural	70.6	84.2	0.92	4.4	92	83	84
Batch	Water	15	Furfural	70.4	83.8	1.14	4.2	90	83	68
Two-stage 9.5 h	Water	15	Furfural	58.2	75.9	1.81	4.3	90	73	37

Either liquid fraction acquired after acid pretreatment or MilliQ water is used to solubilize the solids. As inoculum, either a preculture to which furfural was added was used, or a reference preculture was used which did not contain furfural. Addition of 20% DW bagasse fibres was either done as batch at the start of fermentation, or in two stages of 10% DW. Lactic acid was determined via HPLC

*C*_LA_ concentration of lactic acid at the end of the SSF in g/l

*A*_La_ total lactic acid produced in g

*Q*_v,av_ average volumetric lactic acid productivity in g/l/h

*Q*_v,max_ maximum volumetric lactic acid productivity in g/l/h

*Y*_s/La_ estimated conversion efficiency of sugar monomers to lactic acid in W/W

*Y*_Lc/La_ lactic acid production yield on total lignocellulosic sugars in W/W

^a^Total fermentation time, from inoculation of SSF to reaching final lactic acid concentration

It was found that precultivation in the presence of furfural was beneficial for fermentation processes using acid-pretreated bagasse fibres in an SSF set-up. Therefore, all subsequent SSF experiments were inoculated with *B. coagulans* precultivated in the presence of furfural.

### Batch SSF, solubilizing bagasse fibres with water

Although sufficient lactic acid production, combined with a high conversion yield of lignocellulose to lactic acid, was observed in the batch SSF solubilized with liquid fraction, the average productivity was not yet competitive with the productivity reached on high-grade sugars. This is mainly caused by a long lag phase of approximately 40 h. The liquid fraction contains approximately 20 g of sugar per litre, adding ~5 g of sugars to the reactor, but it also contains 2 g/l of acetic acid, 1 g/l g of furfural and some other by-products in minor concentrations (Table 1). These by-products add up to those present in the pretreated bagasse fibres (solid fraction), and are one of the main challenges when using pretreated lignocellulose in fermentation processes [5]. While the addition of more sugars may be interesting to slightly increase total lactic acid production, there is a risk that the additional amount of inhibiting by-products result in a decrease in productivity. Simultaneously, the liquid fraction can also be fermented in a separate fermentation using more robust microorganisms. Therefore, SSF experiments were performed where the solid fraction was solubilized with milliQ water instead of liquid fraction.

Similar to results observed for batch SSF solubilized with liquid fraction, using furfural-containing precultures as inoculum for batch SSF solubilized with water led to a lag phase reduction of 15 h compared to using a control preculture (data not shown). Still, an extensive lag phase of approximately 32 h was observed at the beginning of the batch SSF solubilized with water (Fig. 2). The production phase, however, showed a high lactic acid productivity, with a maximum lactic acid productivity of 4.2 g/l/h, similar to batch SSF solubilized with liquid fraction.

Batch SSF using solid fraction solubilized with water was performed with two different enzyme concentrations, 7.5% V/DW and 15% V/DW (Fig. 2; Table 2). In both fermentations the sugar concentrations decreased during the production phase and reached levels below 5 g/l after 52 and 55 h for the fermentation with 7.5% V/DW and 15% V/DW enzyme cocktail, respectively (Additional file 1: Fig S1). The fermenter in which 7.5% V/DW of enzymes was used showed a sharp decrease in lactic acid productivity after 52 h to only 0.51 g/l/h, whereas the SSF containing 15% V/DW showed an average productivity of 1.44 g/l/h between 55 and 62 h. At 66 h, 5 ml (2.5% V/DW) additional enzyme cocktail was administered to increase enzyme concentrations to 10% V/DW. Immediately, the glucose concentration increased, reaching a maximum concentration of 5 g/l, and lactic acid production resumed in a similar fashion as was observed for fermentations using 15% V/DW enzymes. This shows that enzymatic hydrolysis of sugars was the rate-limiting step of the batch SSF fermentation containing 7.5% V/DW enzyme cocktail, while 10% V/DW enzymes was a sufficiently high enzyme dosage.

After 68 h, the process containing 15% V/DW enzyme cocktail reached a lactic acid concentration of 70.4 g/l, equivalent to a total lactic acid production of 83.8 g. This corresponds to a lactic acid production yield on total lignocellulosic sugars of 83% W/W and a conversion efficiency for *B. coagulans* of monomeric sugar to lactic acid of 92% W/W. Overall, an average lactic acid productivity of 1.14 g/l/h was reached, which is significantly higher than the average productivity of 0.92 g/l/h observed for batch SSF solubilized with liquid fraction. However, average productivity during batch SSF solubilized with water was still low compared to lactic production on high-grade sugars.

Compared to SHF, the process time of 68 h during batch SSF is short. Where enzyme hydrolysis alone during SSF can take between 48 and 72 h, and the subsequent fermentation requires another 40–60 h [13]. Therefore, although batch SSF using furfural-containing precultures cannot compete with high-grade sugar processes, it is already an interesting alternative to SHF (Table 5). An additional benefit of the batch SSF presented in this study is that a low inoculum size is used, which normally requires high-grade sugars to grow. Furthermore, the enzyme dosage of 10.5–15.8 FPU/g DW that was administered to the batch SSF is relatively low, in most other studies an enzyme dosage of 20–40 FPU/g DW material was used [11, 20, 21]. Enzymatic hydrolysis is considered to be an expensive process step in the conversion of lignocellulose to chemicals due to the high costs of enzyme cocktails combined with high addition [6].

### Two-stage simultaneous saccharification and fermentation (SSF)

Although the maximum productivity in batch SSF was in a similar range as observed for fermentations using high-grade sugars, the average productivity was low still due to a long lag phase (Fig 2, Additional file 1: Fig S1). The end of the lag phase coincided with a reduced viscosity of the culture due to enzymatic hydrolysis, suggesting that the long lag phase was caused by the high viscosity of the broth.

A decrease in viscosity can be achieved by adding the lignocellulosic material in two stages, instead of adding all lignocellulose as batch at the start of the SSF.

Using this approach of a two-stage SSF, the microorganisms directly started to grow without a visible lag phase after inoculation, and a significant lactic acid production was observed within 3 h. After 37 h, a total lactic acid production of 75.9 g was reached with an average lactic acid productivity of 1.81 g/l/h, and a maximum productivity of 4.3 g/l/h (Table 2; Fig. 2). During the first 23 h of the two-stage SSF, an average productivity of 2.54 g/l/h was observed. This productivity is similar to the average productivity of 2.5–3 g/l/h [12–14] (Table 3) which is often seen during fermentation of *B. coagulans* using high-grade sugars as carbon source.

**Table 3:** Different fermentation processes containing high-grade sugars, inoculated with *B. coagulans*

Strain	Carbon source	C_s_ (g/l)	N-source (g/l)	*C*_LA_ (g/l)	*A*_LA_ (g)	*Q*_v,,av_ (g/l/h)	*Q*_v,max_ (g/l/h)	Y_s/LA_(%)	Time (h)^a^	Source
*B. coagulans DSM2314*	High-grade glucose + xylose	72 +24	10 YE, 20 PE	59.3	83	2.50	5.1	92	29	Van der Pol [15]
*B. coagulans DSM2314*	High-grade glucose + xylose	72 +24	10 YE, 2NH_4_PO_4_, 3NH_4_SO_4_	55.6	78.6	2.40	3.9	86	28	Van der Pol [15]
*B. coagulans SIM*-*7*	High-grade glucose	126	25 YE	89.3	–	2.8	5.8	90	31	Michelson [24]

Different nitrogen sources were used: *YE* yeast extract, *PE* peptone

*C*_LA_ concentration of lactic acid at the end of the SSF in g/l

*A*_LA_ total lactic acid produced in g

*Q*_v,av_ average volumetric lactic acid productivity in g/l/h

*Q*_v,max_ maximum volumetric lactic acid productivity in g/l/h

*Y*_s/La_ estimated conversion efficiency of sugar monomers to lactic acid in W/W

*Y*_Lc/La_ lactic acid production yield on total lignocellulosic sugars in W/W

^a^Total fermentation time, from inoculation of SSF to reaching final lactic acid concentration

The average lactic acid productivity in two-stage SSF is much higher than for batch SSF, but total lactic acid production and lactic acid production yields on lignocellulose were slightly lower for two-stage SSF experiments. In previous studies, it is suggested that activity of enzymes present in the cocktail can be repressed by increasing lactic acid concentrations [22, 23]. To test this hypothesis, the activity of GC220 was tested in the presence of high lactic acid concentrations. An experiment to determine initial enzyme activity showed that the presence of 50 g/l of lactic acid resulted in a 51% reduction in glucan monomerization by the enzyme cocktail GC220, whereas the presence of 100 g/l lactic acid results in a full inhibition of the enzymes. Furthermore, it was found that 11% more residual glucans and xylans were present at the end of the two-stage SSF compared to the end of the batch SSF (Table 4). In the batch SSF, a total of 93.4 g of sugar polymers were monomerized, while in the two-stage SSF, 83.2 g of sugar polymers were monomerized. Since the batch SSF shows an extensive lag phase where lactic acid concentrations are low, it can be suggested that the enzymes in batch SSF are less inhibited, leading to an increased monomerization compared to two-stage SSF. Both the batch SSF and the two-stage SSF resulted in similar conversion yield of monomeric sugars to lactic acid of approximately 90% W/W. Therefore, the lower lactic acid titre observed in the two-stage SSF is probably caused by a decreased enzymatic hydrolysis of the lignocellulosic polymers.

**Table 4:** Glucan and xylan monomerization during batch SSF and two-stage SSF, the average lactic acid produced in batch SSF and two-stage SSF, and the conversion yield of lactic acid on monomerized sugars

	Monomerized sugars (g)^a^	La (g)	*Y*_g/La_
Batch SSF 15% V/DW enzymes	93.4	83.8	89.7%
Two-stage SSF 2nd batch T = 9.5	84.2	75.9	90.0%

*La* lactic acid produced at the end of the fermentation in g

*Y*_*g/*LA_ conversion efficiency of sugar monomers to lactic acid in W/W

^a^Sugars: total monomerization of glucan and xylan polymers during SSF

**Table 5:** Production of lactic acid from pretreated lignocellulosic material using either a separate enzymatic hydrolysis and fermentation process (SHF), or a combined process (SSF)

	Microorganism	Feedstock	Chemical pretreatment	SSF/SHF	Detoxification	Enzymatic hydrolysis	EH^a^ (h)	Inoculum (% v/v)	C_LA_ (g/l)	*Y*_Lc/LA_ (%)	*Y*_S/LA_ (%)	*Q*_LA F_ (g/l/h)	*Q*_LA, O_ (g/l/h)	Source
1	*P. acidilactici DQ2*	Corn stover	2.5% H2SO4, 3 m, 190C	SSF	Biological 5 days	Acc 1000 15 FPU/g DW	8	20	101.6	77.2	ND	1.06	0.45	Zhao et al. [25]
2	*Bacillus* sp*. NL01*	Corn stover	3% H2SO4 2 h RT, 5 m 170C, SE	SHF	Centrifugation after EH	Cell 1.5L 15 FPU/g DW + Novo 188 30 CBU/g DW	48	8	61.3	ND	70	1.46	0.67	Ouyang et al. [21]
3	*B. coagulans DSM2314*	Wheat straw	10% NaOH, 16 h, 85C	SSF	None	GC220 18 FPU/g DW	2	20	40.7	43	81	0.74	0.56	Maas et al. [11]
4	*E. faecalis RKY1*	Oak wood	0.5%H2SO4 24 h RT, 5 m 215C, SE	SHF	Centrifugation after EH	Cell 1.5L 15 FPU/g DW + Novo 188 30 CBU/g DW	48	?	93	ND	80	1.7	0.74	Wee et al. [26]
5	*B. coagulans DSM2314*	Sugarcane bagasse + LF	0.72% H2SO4, 15 m, 170C, SE	SSF	None	GC220 13.4 FPU/g DW	5	5	64.1	74	80	0.78	0.72	This article
6	*B. coagulans DSM2314*	Sugarcane bagasse + LF	0.72% H2SO4, 15 m, 170C, SE	SSF	None	GC220 13.4 FPU/g DW	5	5 (Fu P)	74.6	87	94	0.92	0.79	This article
7	*B. coagulans DSM2314*	Sugarcane bagasse	0.72% H2SO4, 15 m, 170C, SE	SSF	None	GC220 15.2 FPU/g DW	4	5 (Fu P)	70.4	83	90	1.14	0.98	This article
8	*B. coagulans DSM2314*	Sugarcane bagasse	0.72% H2SO4, 15 m, 170C, SE	SSF (two-stage)	None	GC220 15.2 FPU/g DW	6	5(Fu P)	58.7	73	90	1.81	1.33	This article

LF *liquid fraction*, *RT room temperature*, *EH* enzymatic hydrolysis. *Acc* accelerase, *Cell* cellulast, *Novo* novozymes. *nd* not determined, *Fu P* furfural preculture, *C*_LA_ concentration of lactic acid at the end of the SSF in g/l, *Y*_Lc/LA_ lactic acid production yield on total lignocellulosic sugars in W/W, *Y*_s/LA_ estimated conversion efficiency of sugar monomers to lactic acid in W/W, *Q*_LA.F_ average volumetric lactic acid productivity during fermentation phase in g/l/h, *Q*_LA.O_ average volumetric lactic acid productivity over the total process in g/l/h, *Fu P* inoculum precultivated in the presence of 1 g/l furfural

^a^Enzymatic hydrolysis time required during SHF, or pre-hydrolysis time prior to inoculation during SSF

## Conclusions

Production of lactic acid from acid-pretreated sugarcane bagasse was performed with two different processes, a batch SSF and a two-stage SSF. In these processes, enzyme hydrolysis and fermentation were combined for shorter process times compared to SHF. The objective of this study was to reach a lactic acid productivity in SSF experiments using lignocellulose similar to fermentations using high-grade sugars as feedstock.

It was shown that addition of furfural to the preculture reduced the initial growth lag phase and increased lactic acid production. In batch SSF, up to 91.7 g of lactic acid could be produced, with a conversion yield of monomeric sugars to lactic acid of 94% W/W, and a lactic acid production yield on total sugars present in pretreated bagasse of 87% W/W. Productivities during batch SSF of 0.78–1.14 g/l/h still low compared to productivities of 2.5–3 g/l/h reached on high-grade sugars.

In two-stage SSF, viscosity of the fermentation broth was reduced. This results in an average productivity over the total process of 1.81 g/l/h. During the first 23 h of fermentation, an average lactic acid productivity of 2.54 g/l/h was observed. This productivity is similar to the productivity obtained in fermentation process using high-grade sugars as feedstock.

When the SSF processes as proposed in this research are compared to other processes, it can be concluded that adequate production of lactic acid from lignocellulose was successfully accomplished by an SSF process which uses a combination of acid-pretreated bagasse, *B. coagulans* as microorganism and GC220 as enzyme cocktail (Table 5). Furthermore, an improvement of enzymatic hydrolysis at high lactic acid concentrations will further increase the competitiveness of the SSF process in comparison to using high-grade sugars.

## Additional file

**Additional file 1: Fig S1.** Sugar concentration over time during fermentation. Simultaneous Saccharification and Fermentation (SSF) experiments performed using 200 g/l acid pretreated bagasse DW and 10.5-15.8 FPU/g DW bagasse GC220 enzyme cocktail in pH controlled fermentations with a start volume of 1 litre. As inoculum, either furfural containing or reference preculture was used. The bagasse was either added in one batch or in two stages. Glucose was measured using HPLC. The following experiments were performed: ●: batch SSF solubilised with liquid fraction, inoculated with reference preculture, ■: batch SSF solubilised with liquid fraction, inoculated with furfural preculture, ▼: batch SSF solubilised with water, inoculated with furfural preculture (15% V/DW enzymes), ◆: two-stage SSF solubilised with water, inoculated with furfural preculture.
