# Supplementary material for: Production of l(+)-lactic acid from acid pretreated sugarcane bagasse using Bacillus coagulans DSM2314 in a simultaneous saccharification and fermentation strategy
Source: Biotechnol Biofuels. 2016 Nov 15;9:248. doi: 10.1186/s13068-016-0646-3 (PMC5111225; doi:10.1186/s13068-016-0646-3)
Supplement: Supplementary file 1 — Additional file 1: Fig S1. Sugar concentration over time during fermentation. Simultaneous Saccharification and Fermentation (SSF) experiments performed using 200 g/l acid pretreated bagasse DW and 10.5-15.8 FPU/g DW bagasse GC220 enzyme cocktail in pH controlled fermentations with a start volume of 1 litre. As inoculum, either furfural containing or reference preculture was used. The bagasse was either added in one batch or in two stages. Glucose was measured using HPLC. The following experiments were performed: ●: batch SSF solubilised with liquid fraction, inoculated with reference preculture, ■: batch SSF solubilised with liquid fraction, inoculated with furfural preculture, ▼: batch SSF solubilised with water, inoculated with furfural preculture (15% V/DW enzymes), ◆: two-stage SSF solubilised with water, inoculated with furfural preculture. [file 13068_2016_646_MOESM1_ESM.docx]

**Supplementary figure 1: sugar concentration over time during fermentation**


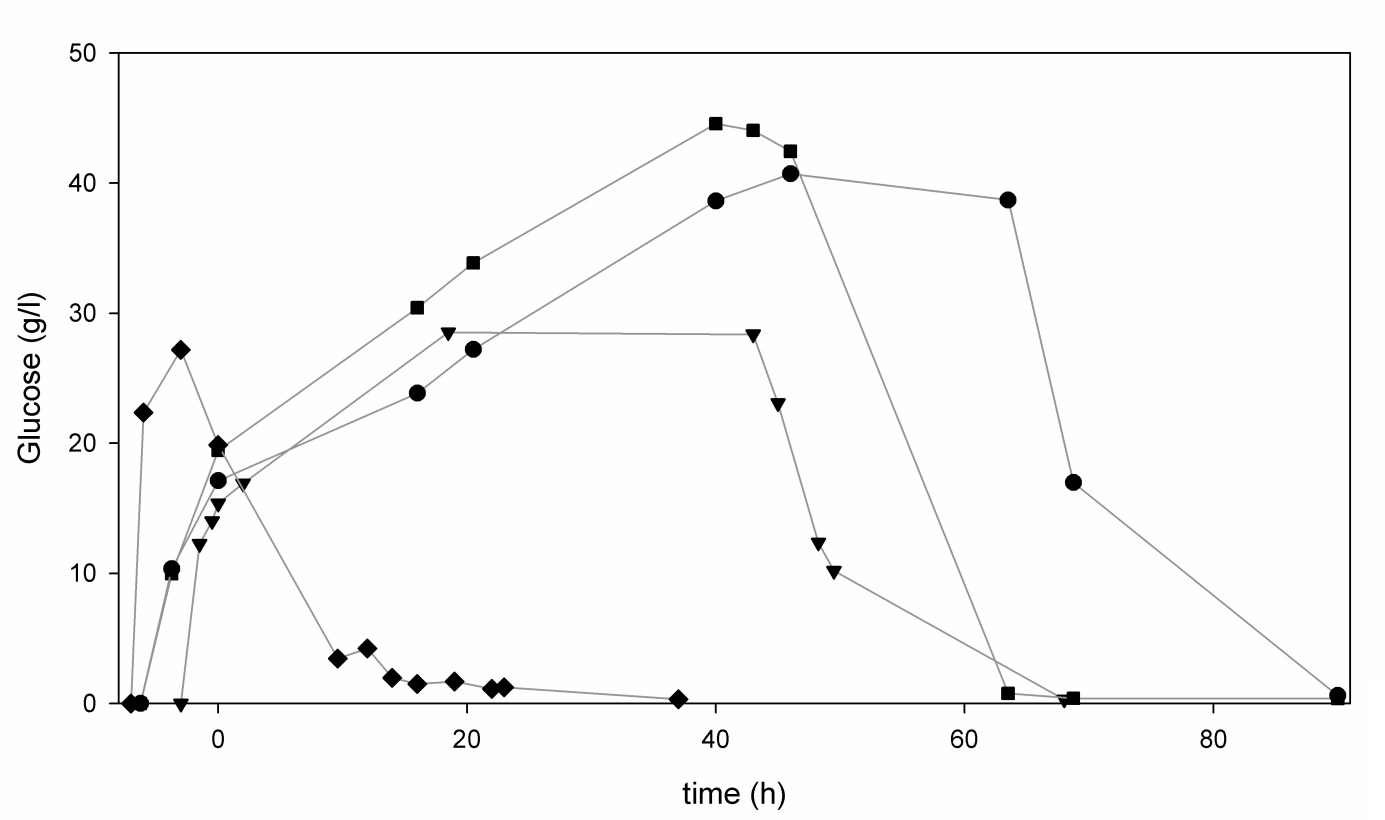


*Figure A: Simultaneous Saccharification and Fermentation (SSF) experiments performed using 200 g/l acid pretreated bagasse DW and 10.5-15.8 FPU/g DW bagasse GC220 enzyme cocktail in pH controlled fermentations with a start volume of 1 litre. As inoculum, either furfural containing or reference preculture was used. The bagasse was either added in one batch or in two stages. Glucose was measured using HPLC. The following experiments were performed:* ⚫: *batch SSF solubilised with liquid fraction, inoculated with reference preculture,* ■: *batch SSF solubilised with liquid fraction, inoculated with furfural preculture,* ▼: *batch SSF solubilised with water, inoculated with furfural preculture (15% V/DW enzymes),* ⯁: *two-stage SSF solubilised with water, inoculated with furfural preculture.*
